# Rapid test for *Mycobacterium leprae* infection: a practical tool for leprosy

**DOI:** 10.1186/s40249-024-01262-9

**Published:** 2024-12-02

**Authors:** Louise Pierneef, Anouk van Hooij, Danielle de Jong, Gaby Wassenaar, Els Verhard, Elisa Tjon Kon Fat, Nadine Engel, Marufa Khatun, Santosh Soren, Abu Sufian Chowdhury, Colette van Hees, Paul Corstjens, Annemieke Geluk

**Affiliations:** 1 Leiden University Center of Infectious Diseases, Leiden University Medical Center, Leiden, The Netherlands; 2 Department of Cell and Chemical Biology, Leiden University Medical Center, Leiden, The Netherlands; 3 Rural Health Program, The Leprosy Mission International Bangladesh, Nilphamari, Bangladesh; 4 Department of Dermatology, Erasmus Medical Center, Rotterdam, The Netherlands

**Keywords:** *Mycobacteriumleprae*, Diagnosis, Lateral flow, Leprosy, IgM, Quantitative UCP-based rapid test, Target product profile, Upconverting reporter particle

## Abstract

**Background:**

Detection of infection with *Mycobacterium leprae* allows timely prophylactic treatment, thereby reducing transmission as well as the risk of permanent, leprosy-associated nerve damage. However, since there is no worldwide-implemented standard test for *M. leprae* infection, detection of infection in asymptomatic individuals remains a major challenge for control programs in endemic areas. In previous studies, we developed and field-tested a lateral flow assay (LFA) quantitatively detecting human IgM against *M. leprae*-specific phenolic glycolipid I (anti-PGL-I), a marker for both active and past infection. This rapid test utilizes luminescent, background-free, up-converting reporter particles (UCP) and immunochromatography (i.e. the UCP-LF test platform) for accurate quantitation of anti-PGL-I IgM without operator bias. The aim of this study was to evaluate the final version of this quantitative UCP-based rapid test (i.e. PGL-I QURapid), using serum and fingerstick blood (FSB).

**Methods:**

The test comprises a lateral flow strip, in a standard plastic or biodegradable cassette. It can be provided with a humanized, recombinant control to monitor test performance and calculate accurate anti-PGL-I IgM levels. The performance of this QUR-test was assessed using serum and FSB from patients with leprosy *(n* = 214), tuberculosis (*n* = 20), buruli ulcer (*n* = 19), leishmaniasis (*n* = 14), non-tuberculous mycobacterial (*n* = 35) infections, as well as healthy Dutch individuals (*n* = 710) and humanized, recombinant anti-PGL-I IgM antibodies. Plot receiver operating characteristic curves were created and sensitivity (Sn), specificity (Sp) and the area under the curve were calculated to evaluate test performance.

**Results:**

Test results classified multibacillary leprosy patients with 95.0% Sn and 100% Sp using serum and 91.5% Sn and 99.8% Sp using FSB. Qualitative test results could be read after 2 min flow time, with accurate quantitation from 10 min onwards. The new anti-PGL-I IgM control supports production of batches with predetermined seropositivity thresholds and monitoring of the PGL-I QUR-test in various settings.

**Conclusion:**

The operational version of the PGL-I QURapid with point-of-care applicability, meets the WHO target product profile criteria. Thus, this QUR-test is ready for public health implementations.

**Graphical Abstract:**

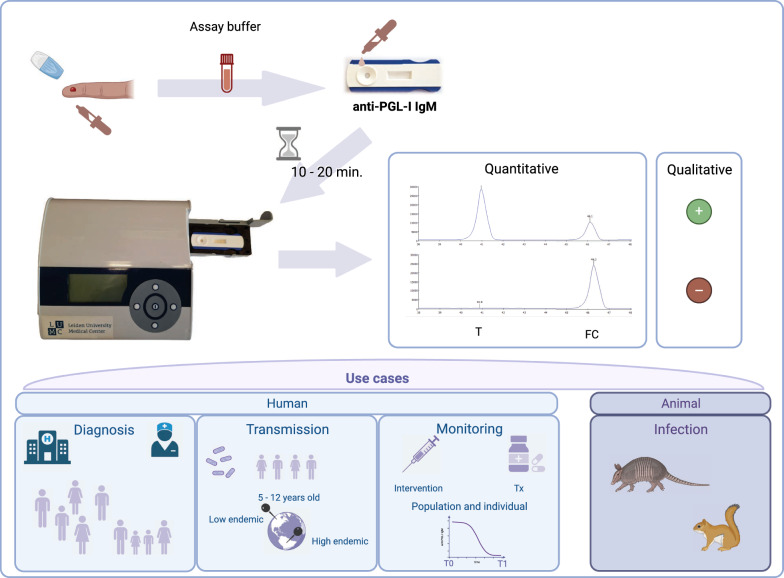

**Supplementary Information:**

The online version contains supplementary material available at 10.1186/s40249-024-01262-9.

## Background

Leprosy is a neglected tropical disease (NTD) caused by *Mycobacterium leprae* or *M. lepromatosis* affecting the skin and the peripheral nerves [1–3]. Despite the available multidrug therapy (MDT)—which can effectively kill *M. leprae* when treatment is started in time—leprosy remains a significant health problem in endemic areas where about 200,000 new cases are reported annually from over 120 countries [4, 5]. The World Health Organization (WHO)’s Global Leprosy Strategy 2021–2030 aims to significantly reduce the number of new cases with grade 2 disability and new child cases by focusing on early detection of disease and interruption of transmission [6]. To achieve the latter, it is vital to identify and prophylactically treat *M. leprae* infected individuals (to prevent progression to leprosy disease) as well as therapeutically treat leprosy patients [7, 8].

Leprosy diagnosis is still dependent on the identification of clinical symptoms, and studies have shown that health practitioners lacking sufficient experience in clinical leprosy, may not recognize disease symptoms [5, 9]. In addition, detecting *M. leprae* infection in individuals without clinical symptoms remains a major challenge for control programs in endemic areas. In this respect, the use of different tests to detect infection, in particular variable analyses, assays, or qualitative measurements only, furthermore impedes comparison of *M. leprae* infection rates globally [10]. The most severe form of leprosy (multibacillary) is characterized by numerous antibodies [11], in particular immunoglobulin M (IgM), whereas IgG and IgA are detected mostly at lower levels and in patients only [12]. Blood levels of IgM against *M. leprae*-specific phenolic glycolipid I (anti-PGL-I), correspond to an individual’s bacterial load [11], thereby allowing detection of *M. leprae* infection as well as treatment monitoring of MB leprosy patients [11, 13, 14]. However, most PB patients are able to kill *M. leprae* efficiently and therefore do not mount antibody responses directed against PGL-I [1, 11, 15–17]. Furthermore, besides detecting infection with *M. leprae*, anti-PGL-I antibodies were also detected in leprosy patients and red squirrels infected with *M. lepromatosis* (unpublished data and [18]).

Since *M. leprae* infection in young children is recent by definition, anti-PGL-I seroprevalence in healthy young children could be a proxy of transmission in a population [10, 19]. Therefore, the WHO Task Force on definitions, criteria and indicators for interruption of transmission and elimination of leprosy recommends to consider using anti-PGL-I seroprevalence in children as a criterion for monitoring (interruption of) transmission in areas aiming at elimination of leprosy [20]. Additionally, seroprevalence has potential to assess the effect of interventions like post-exposure prophylaxis at population as well as individual level. For all applications, quantitative assessment of anti-PGL-I serology is essential. Thus, availability of a collectively acceptable user-friendly test would facilitate strategies to uniformly measure infection.

Given the fact that leprosy particularly affects poor communities in low-resource settings, a rapid, low-complexity, field-friendly diagnostic test is needed to identify infected individuals. Other tests developed so far, are either qualitative or not rapid (Supplementary Table 1) [11, 21–25]. Therefore, we have developed a robust, user-friendly test to quantitatively detect anti-PGL-I IgM using up-converting phosphor (UCP) technology in a low-cost LFA format [11, 16, 17, 26, 27]. Previously, the anti-*M. leprae* PGL-I UCP-LFA (PGL-I UCP-LFA) has been used in a serosurvey among 1857 children in India [19] where it was well-accepted for field work. Currently, this quantitative UCP-based rapid test (i.e. PGL-I QURapid) is applied in a clinical trial in Bangladesh (NCT06222372: 03/01/2024; https://clinicaltrials.gov/study/NCT06222372) and evaluated in ongoing field studies in Brazil, Bolivia, Indonesia, Nepal, and Madagascar. The basis of the UCP-LFA is formed by ultrasensitive reporter particles [28], which, upon excitation by IR light, up-convert the energy to give a visible 550-nm green emission. Since no biological specimen in nature up-converts low-energy IR light, UCP applications are unaffected by specimen background and display excellent signal-to-noise ratios [29]. This QUR-test does not require sophisticated analytical laboratory equipment or elaborate staff training and portable battery-operated readers provide full instrument-assisted analysis. In view of field and point-of-care/point-of-contact (POC) applications, the assay utilizes a minimally invasive fingerstick blood (FSB) sample. The PGL-I QURapid allows convenient storage at ambient temperature and worldwide shipping without the need for a cold chain.

Here, we analyze serum and FSB samples from patients with leprosy. For specificity purposes, sera from patients with other mycobacterial infections, such as tuberculosis (TB) or buruli ulcer (BU), as well as non-tuberculous mycobacterial (NTM) infections were similarly assessed. Also, as leishmaniasis is included in the differential diagnosis of leprosy [30] and as both diseases overlap geographically, sera of leishmaniasis patients were included.

For the optimization of the performance of any quantitative test, the determination of the threshold for positivity is essential. Therefore, the optimally required sensitivity (Sn)/specificity (Sp) as defined in a target product profile (TPP) published by the WHO in 2023 were used as initial targets for this study (ideal: ≥ 94% Sn; ≥ 99.9% Sp) [31]. Since a robust quality control is vital for monitoring test performance, humanized, recombinant anti-PGL-I IgM was developed, to allow normalization of threshold values between batches of the PGL-I QURapid.

We present an operational QUR-test for the detection of *M. leprae* infection which can be made available for research and large-scale population studies and clinical trials. This study aims to describe the performance of this new QUR-test in detail, regarding its optimal flow time, reproducibility, robustness, sensitivity and specificity. Importantly, the PGL-I QURapid can be used for different applications and populations, providing a significant tool for multiple use cases in the leprosy field worldwide.

## Methods

### Samples

Biobanked samples were derived from the following study groups (Table 1).

**Table 1:** Characteristics of participants’ samples

Group	Country of sampling	*n*	Age, years, mean (range)	Sex (% female)	Refs.
Leprosy patients	Bangladesh	209	38 (8–83)	20	[13, 17, 32, 33]
	The Netherlands	5	46 (24–64)	60	[26]
TB patients	The Netherlands	20	44 (15–84)	20	[35, 36]
Leish patients	The Netherlands	7	59 (24–82)	29	[37]
	The Netherlands	7	na	na	[38]
BU patients	Ghana	19	13*	59	[39]
NTM patients	The Netherlands	35	30 (1–72)	56	[41–44]
Healthy controls	The Netherlands	710	42 (18–67)	72	[45]

Overview of the different samples tested including country of sampling, number of individuals, age mean (range), and sex (% female)

*BU* buruli ulcer, *Leish* leishmaniasis, *na* not available, *NTM* non-tuberculous mycobacterial infection, *TB* tuberculosis

*Median

### Leprosy patients

Serum/plasma and FSB samples from newly diagnosed leprosy patients recruited between June 2013 and May 2022 in leprosy endemic areas in the Northwest of Bangladesh [13, 17, 32, 33], were tested. Patients were diagnosed according to the National Leprosy Control Program [32]. Patients with five or fewer skin lesions were grouped as paucibacillary (PB), whereas patients with more than five skin lesions were grouped as multibacillary (MB) leprosy. In this study, PB patients with a bacterial index (BI) of 0 (*n* = 76) and MB patients with a positive BI (1–6; *n* = 133) were included. From leprosy patients recruited in the Netherlands (*n* = 5), FSB samples were collected on a voluntary basis between January 2020 and February 2023 at the Department of Dermatology, Erasmus MC, University Medical Center (EMC), Rotterdam, the Netherlands [26]. Leprosy in the Netherlands was diagnosed based on clinical examination, histopathology of skin biopsies and PCR of skin biopsies, using anti-PGL-I serology and microbiological testing as adjunct diagnostic tools. Additionally, leprosy histology of biopsies was applied for classification according to Ridley and Jopling [34].

### TB patients

Serum samples from TB patients (*n* = 20) during or after treatment, recruited between January 2002 and January 2003 at the Leiden University Medical Center (LUMC), Leiden, the Netherlands [35, 36] were included.

### Leishmaniasis patients

Serum samples of leishmaniasis patients were either sent to the Clinical Microbiological Laboratory of the Department of Medical Microbiology, LUMC, Leiden, the Netherlands, for routine leishmaniasis diagnostic testing (*n* = 7) [37] or collected from PCR- and/or microscope-confirmed leishmaniasis patients at the Department of Medical Microbiology and Infectious Diseases, EMC, Rotterdam, the Netherlands (*n* = 7) [38].

### Buruli ulcer (BU) patients

Serum samples from BU patients (*n* = 19) were obtained from the Swiss Tropical and Public Health Institute, Allschwil, Switzerland, and collected from villages within the Obom subdistrict of the Ga-South district of Ghana [39]. *IS2404* PCR was used to confirm infection with *Mycobacterium ulcerans*. Lesions were classified following the WHO classification [40].

### Patients infected with non-tuberculous mycobacteria (NTM)

Sera from individuals with various NTM infections (*n* = 35) were collected between March 2000 and March 2003 at the Department of Infectious Diseases, LUMC, Leiden, the Netherlands [41–44].

### Healthy controls

FSB (*n* = 500) and serum (*n* = 115) samples of health care workers from three hospitals in the Netherlands (LUMC, Radboud University Medical Center and University Medical Center Utrecht) who participated on a voluntary basis in a BCG-vaccination trial during the COVID-19 pandemic [45], were selected as non-endemic controls (NEC). This included an even division of samples derived from either BCG- or placebo-vaccinated health care workers 12 weeks after vaccination. In addition, serum samples from Dutch healthy blood bank donors (*n* = 95) were tested.

### Quality control samples

As part of the quality control for the PGL-I QURapid, sera of clinically-diagnosed leprosy patients were selected based on their anti-PGL-I IgM levels in standard anti-PGL-I IgM ELISAs [11, 21]: anti-PGL-I IgM highly seropositive (High; *n* = 2), medium (Med; with an OD around the cut-off for seropositivity in ELISAs; *n* = 1). Seronegative (Neg; *n* = 2) samples included were from healthy Dutch blood bank donors without travel history to leprosy endemic areas. These control samples were also used to assess intra- and inter-operator variability of the assay. “Inter-operator variation” was here defined as the amount of variation between the results obtained by three operators testing the same sample using the PGL-I QURapid (each preparing their own dilutions). “Intra-operator variation” was referred to as the amount of variation in the test results when one operator tested the same samples more than once (e.g. replicates in the same experiment and over multiple days). Anti-PGL-I IgM highly seropositive, medium and seronegative control sera and FSB samples were analyzed at 2, 5, 10, 20, 60 min and 24 h after sample addition to determine the effect of time after start of sample flow until measurement for the PGL-I QURapid. As a control, humanized, recombinant anti-PGL-I IgM produced by hybridoma technology (Eurogentec, Seraing, Belgium; stock concentration: 1 mg/ml) was used.

### PGL-I QURapid

Individually packaged UCP-LFA cassettes for detection of human anti-PGL-I IgM antibodies were produced by MaximBio (Rockville, MD, USA) as described previously [19]. The air-tight pouches with test cassettes contained silica dry packs allowing extended shelf life and protection against humidity. The Test (T) line on the LF strip (nitrocellulose membrane; Sartorius UniSart CN95) comprised 100 ng of synthetic PGL-I, phenolic trisaccharide functionalized with a hexanoic acid linker for conjugation to BSA (NPT1-H-BSA; Leiden, the Netherlands [21]). The flow control (FC) line comprised 100 ng rabbit anti-goat IgG (G4018; Sigma-Aldrich, Inc., St. Louis, MO, USA). Goat IgG specific for anti-human IgM (I0759; Sigma-Aldrich, Inc., St. Louis, MO, USA) was conjugated to polyacrylic acid functionalized UCPs [200 nm, NaYF4:Yb3+, Er 3+; Intelligent Material Solutions Inc. (IMS); Princeton, NJ, USA MS] according to previously described protocols at a concentration of 50 μg antibody per mg UCP [16]. Stock solutions were kept at 4 °C until use. To dry the UCPs onto the glass fiber conjugate-release pad, the material was diluted in a buffer containing 100 mmol/L Tris pH 8.0, 270 mmol/L NaCl, 10% (w/v) sucrose, 1% (w/v) BSA, 0.5% Tween-20, and striped at a density of 100 ng/mm. Components were mounted on plastic backing cards which were cut into LF strips of 4.8 mm width by 6 cm length, added to an appropriate cassette, and individually sealed in a pouch together with a silica dry pack.

### UCP-LFA

50 µl of the 50-fold diluted FSB or serum/plasma sample or diluted recombinant anti-PGL-I IgM stock was added to the test to initiate LF. QUR-tests were analyzed using a battery operated UCP-adapted portable lightweight standalone reader (ESEQuant LFR adapted for UCP; DIALUNOX, Stockach, Germany). Results were calculated as the ratio value (R) between T line and FC line signal based on relative fluorescence units (RFUs) measured at the respective lines.

### Statistical analysis

The statistical software GraphPad Prism version 9.0.1 for Windows (GraphPad Software, San Diego, CA, USA) was used to perform statistical analysis. Mann–Whitney U and Kruskal–Wallis tests were performed to determine the statistical significance between two and three independent groups, respectively. Plot receiver operating characteristic (ROC) curves were created and Sn, Sp and the area under the curve (AUC) were calculated to evaluate test performance. According to analyses described in our previous research on UCP-LFAs [16, 17, 33, 46], the Youden’s index [47] was used to assess cut-offs for anti-PGL-I IgM seropositivity in serum/plasma and FSB samples for this QUR-test batch. However, the WHO TPP published in 2023 [31] was leading in terms of required minimum Sn/Sp for establishing a cut-off. An indecisive range was determined by calculating a lower-specificity cut-off (average of a set of negative controls + 2 × SD) and a high-specificity cut-off (the highest of a set of negative controls + 2 × SD) using R-values below the 99th percentile [48].

### Ethics

Ethical permission for leprosy patient samples was received from the national Research Ethics Committee (Bangladesh Medical Research Council) in Bangladesh (Ref no. BMRC/NREC/2010–2013/1534) [15] and local ethical boards in the Netherlands (MEC-2012-589). Anonymized use of residual serum samples of leishmaniasis patients for scientific purposes was granted by the Medical Ethics Review Board of the EMC, Rotterdam, the Netherlands (MEC 2012-047 and MEC-2015-306) and institutional review board (IRB) of the LUMC, Leiden, the Netherlands (B21.048). The biomaterial and associated clinical data of donors collected in the LUMC healthy voluntary donor service (LuVDS) are released for research purposes only, after being approved by the IRB. The LuVDS Biobank is stored in, and direct use coordinated by, the central biobanking facility at the LUMC, Leiden, the Netherlands. The LUMC Biobank facility was implemented by the LUMC Executive Board as part of the university research infrastructure and acts as a separate entity, servicing all departments of the university medical center. Use of residual serum samples of TB, NTM and BU patients for scientific purposes was approved by the Medical Ethics Committee and IRB of the LUMC (METC project nr: P07.048 & P207/99; TB and NTM patients), and the institutional review board of the Noguchi Memorial Institute for Medical Research (Federalwide Assurance number FWA00001824; BU patients) in Ghana, respectively. The BCG-CORONA trial was registered at clinicaltrials.gov (identifier: NCT04328441) and the Dutch Trial Registry (trialregister.nl, identifier Trial NL8477) and the study protocol was approved by the IRB of the LUMC (NL73249.041.20). All donors gave broad consent.

## Results

### Determining the optimal flow time

**Fig. 1:**
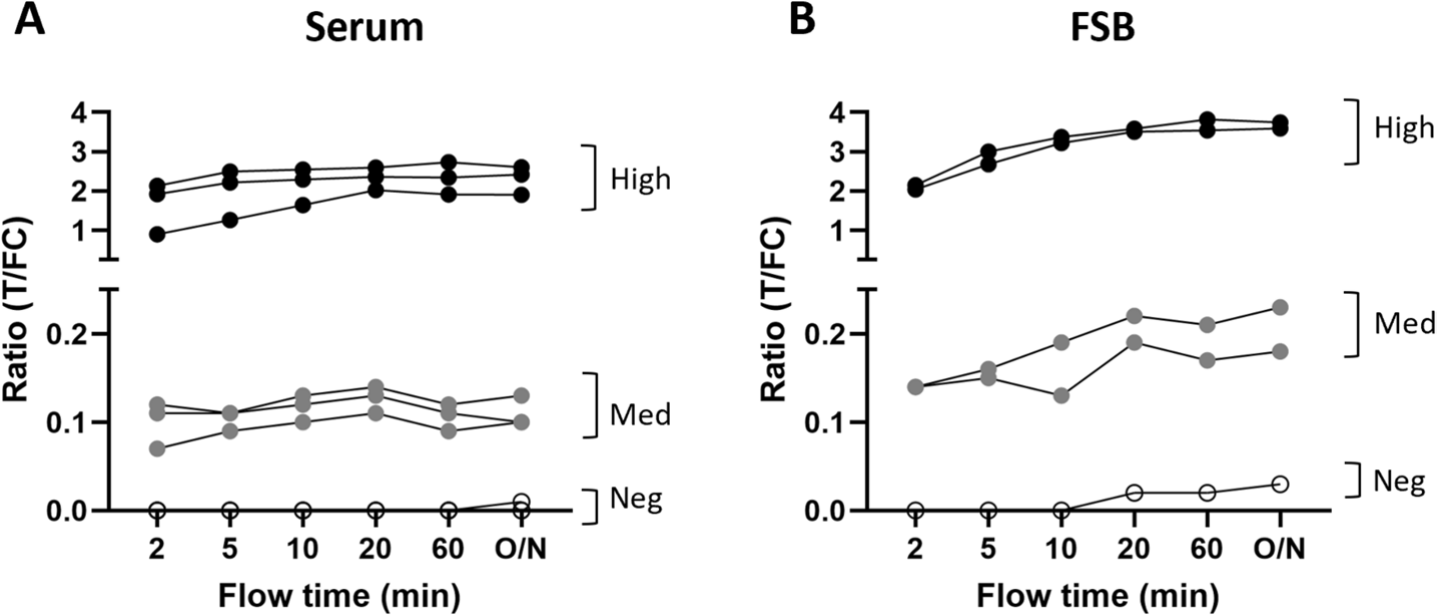
Scanning of PGL-I QURapid by Quant LFR over sample flow time. Anti-PGL-I IgM highly seropositive (High; black dots), medium (Med; grey dots) and seronegative (Neg; open dots) control sera (**A**) and FSB samples (**B**) were assessed using the PGL-I QURapid. Samples were measured at 2, 5, 10, 20, 60 min and 24 h (O/N) after sample addition. Ratio (R-)values (Y-axis) were calculated by dividing the peak area of the test line (T) by the peak area of the flow control line (FC)

Since most leprosy cases occur in remote and/or resource-limited areas, a diagnostic tool for detection of *M. leprae* infection, should be of low-complexity nature such that it is easily implementable in field settings. The here described PGL-I QURapid can (in addition to serum/plasma) be performed with FSB, an easily obtained, low-invasive bio-sample. Since the WHO TPP for an optimal diagnostic test for *M. leprae* infection [31] requires a sample-to-result time of maximum 30 min, we determined the effect of time after start of sample flow until measurement for the PGL-I QURapid using either serum or FSB. Already after 10–20 min, sufficiently stable R-values were observed for both serum (Fig. 1A) and FSB (Fig. 1B) quality control samples that did not vary significantly from values obtained the next day (for T and FC lines see Supplementary Fig. 1). Thus, 10–20 min would be the preferred minimum time after sample addition until scanning of the PGL-I QURapid. Of note is that the strips are stable at ambient temperature and therefore, QUR-tests can be scanned at any later point in time which is convenient for checking data at a central facility.

### Determining test performance

**Fig. 2:**
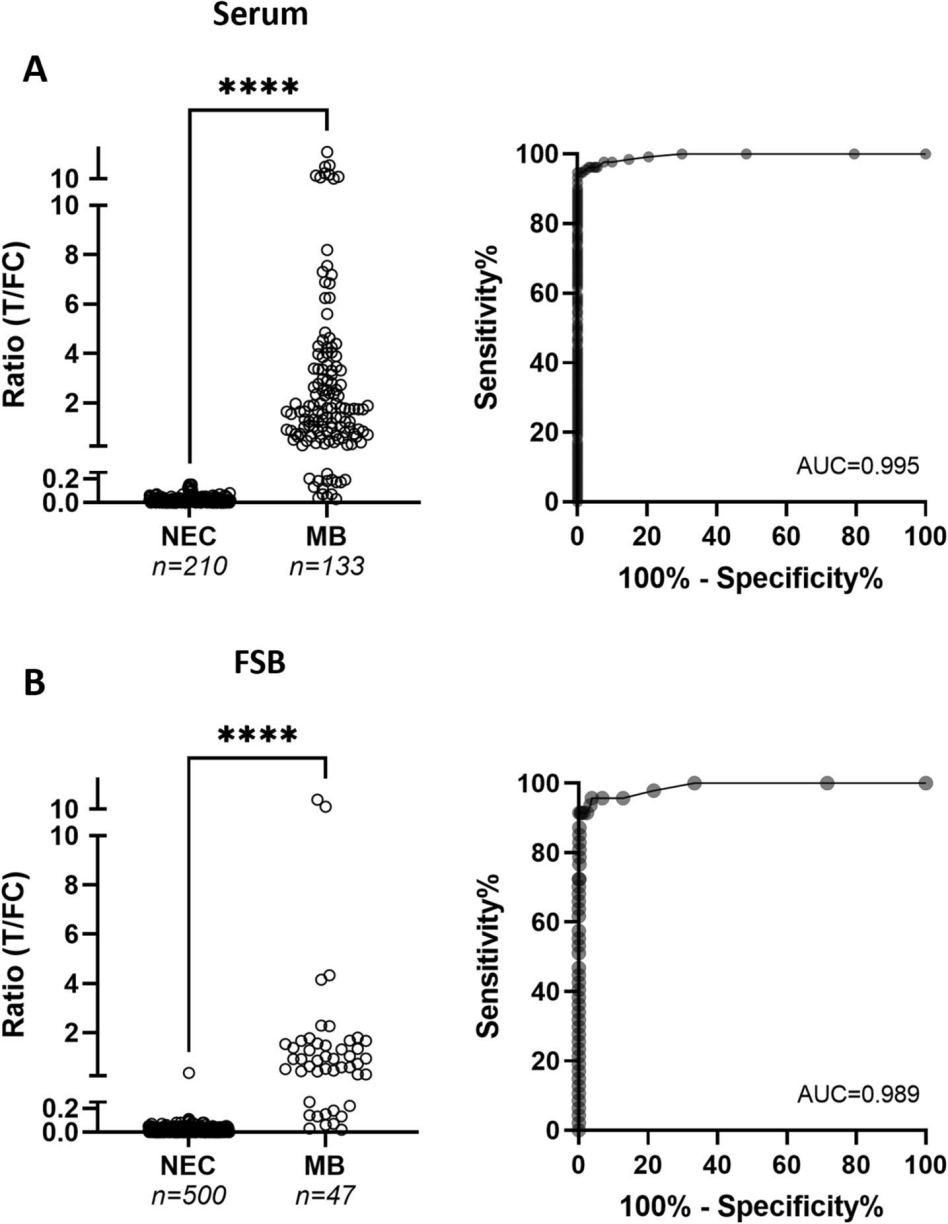
Anti-PGL-I IgM in NEC and MB leprosy patients. Serum samples (**A**) of NEC (*n* = 210) and MB leprosy patients (*n* = 133) and FSB samples (**B**) of NEC (*n* = 500) and MB leprosy patients (*n* = 47) were examined using the PGL-I QURapid. Left panels: R-values for anti-PGL-I IgM in NEC and MB leprosy patients; right panels: corresponding ROC curves. R-values (Y-axis) were calculated by dividing the peak area of the test line (T) by the peak area of the flow control line (FC). A Mann–Whitney U test was performed to determine the statistical significance between the two groups (****P ≤ 0.0001). *AUC* Area Under the Curve, *NEC* non-endemic controls, *MB* multibacillary, *ROC* receiver operating characteristic

To determine test performance, results in sera from MB leprosy patients from Bangladesh (*n* = 133) and healthy adults from the Netherlands (NEC; *n* = 210) were compared. As (previous) infection with *M. leprae* in endemic areas can never completely be excluded, we selected Dutch individuals as control group, since leprosy is an import disease in the Netherlands and there have not been any autochthonous cases for centuries. Test sensitivity was determined based on the requirement to detect clinically diagnosed, BI-positive leprosy patients. R-values, the quantitative test outcome of UCP-LFAs, were significantly higher for MB leprosy patients compared to NEC (Fig. 2A; *P* < 0.0001; AUC: 0.995). Using the Youden’s index, the cut-off value to discriminate clinically diagnosed MB leprosy patients from NEC, was R ≥ 0.160 with a corresponding Sn of 95% (95% *CI:* 89.5–97.4) and Sp of 100% (95% *CI:* 98.2–100) (Supplementary Table 2), meeting the WHO TPP for a test for *M. leprae* infection (ideal: ≥ 94% Sn; ≥ 99.9% Sp). When using the lower-specificity cut-off (R = 0.08; Sn: 96%; Sp: 95%) and the high-specificity cut-off (R = 0.19; Sn: 92%; Sp: 100%), an indecisive range was determined as: R-values between 0.08 ≤ R ≤ 0.19. As the cut-off determined by the Youden’s index, R ≥ 0.16, fell inside this range and also met the WHO TPP criteria, this was considered appropriate for use as the cut-off for seropositivity in serum.

For FSB, test performance for this batch of the PGL-I QURapid was determined by comparing samples collected from 500 NEC and 47 untreated MB leprosy patients (Fig. 2B). Similar to results obtained with sera, R-values were significantly increased for MB leprosy patients compared to NEC (*P* < 0.0001; AUC: 0.989). The cut-off best corresponding to the optimal WHO TPP (≥ 94% Sn; ≥ 99.9% Sp), R ≥ 0.12, resulted in a Sn of 91.5% (95% *CI:* 80.1–96.6) and Sp of 99.8% (95% *CI:* 98.9–100; Supplementary Table 3). When using the lower-specificity cut-off (R = 0.05; Sn: 96%; Sp: 96%) and the high-specificity cut-off (R = 0.12; Sn: 91%; Sp: 100%), an indecisive range was determined for R-values between 0.05 and 0.12. Placing Sp at 100%, R ≥ 0.12 was concluded as appropriate cut-off for seropositivity in FSB.

### Inter- and intra-operator variation

**Fig. 3:**
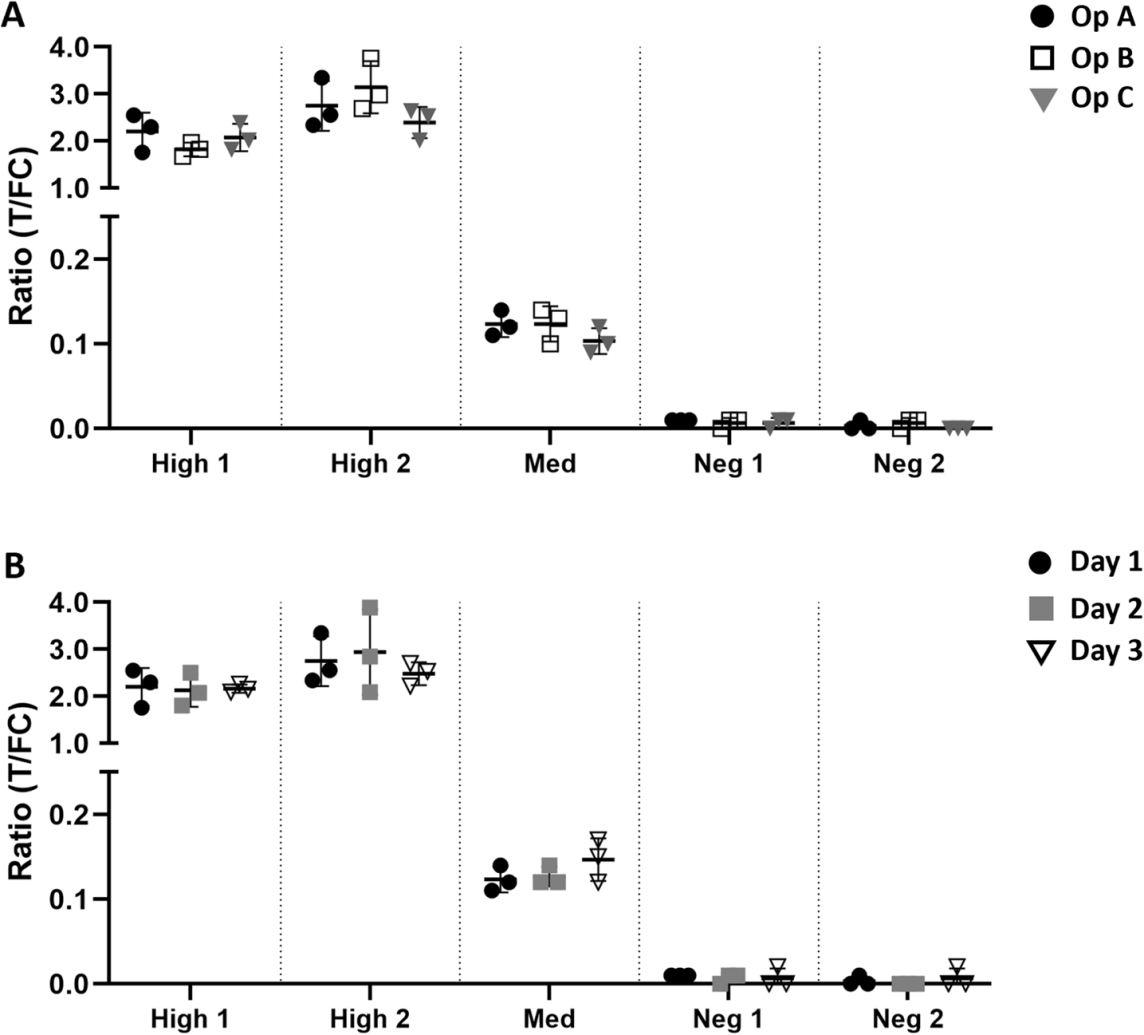
Inter- and intra-operator differences for the PGL-I QURapid. Two anti-PGL-I IgM highly seropositive (High 1–2), one medium (Med) and two seronegative (Neg 1–2) serum samples were examined by the PGL-I QURapid by three different operators (**A**) on three different days (**B**). Each sample was tested in triplicate. Ratio (R-)values (Y-axis) were calculated by dividing the peak area of the test line (T) by the peak area of the flow control line (FC). Mean and SD are shown. Two example figures are shown here. See Supplementary information for complete data. **A** black dots: Operator A (Op A); open squares: Operator B (Op B); grey triangles: Operator C (Op C). **B** Black dots: Test day 1; grey squares: Test day 2; open triangles: Test day 3

To evaluate assay robustness, two anti-PGL-I IgM highly seropositive, one medium and two seronegative control serum samples were tested in triplicate by three different operators. This multi-operator comparison showed that inter-operator differences were not detectable or very low (Fig. 3A; Supplementary Table 4). In addition, reproducibility (intra-operator variation) was evaluated by testing the same five control serum samples on three QUR-tests by each operator on three different days (Fig. 3B; Supplementary Table 4). Day-to-day as well as triplicate differences on the same day were minor (Supplementary Table 4), confirming excellent assay reproducibility.

### Specificity for *M. leprae* infection in the context of other (mycobacterial) infections

**Fig. 4:**
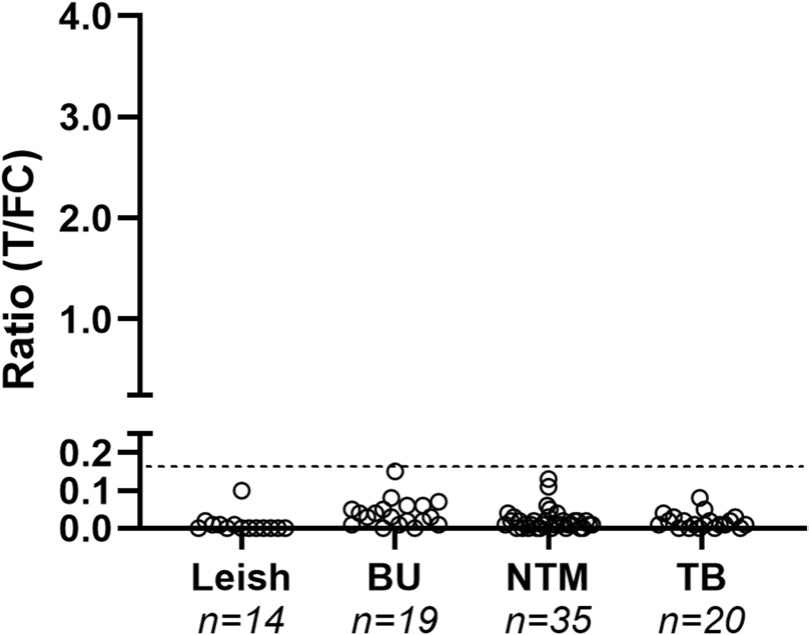
Anti-PGL-I IgM in individuals with leishmaniasis, BU, NTM infections and TB. Serum samples of leishmaniasis (Leish; *n* = 14), buruli ulcer (BU; *n* = 19), non-tuberculous mycobacterial infection (NTM; *n* = 35), and tuberculosis (TB; *n* = 20) patients were examined using the PGL-I QURapid. Ratio (R-) values (Y-axis) were calculated by dividing the peak area of the test line (T) by the peak area of the flow control line (FC). The dotted line represents the cut-off for seropositivity (R ≥ 0.16) in serum

To evaluate assay specificity, serum samples of leishmaniasis (Leish; *n* = 14), BU (*n* = 19), NTM infection (*n* = 35), and TB (*n* = 20) patients from a European area non-endemic for leprosy were tested. Applying a cut-off value of R ≥ 0.16, all leishmaniasis, BU, NTM and TB patients were seronegative for anti-PGL-I IgM (Fig. 4).

### Bacterial load

**Fig. 5:**
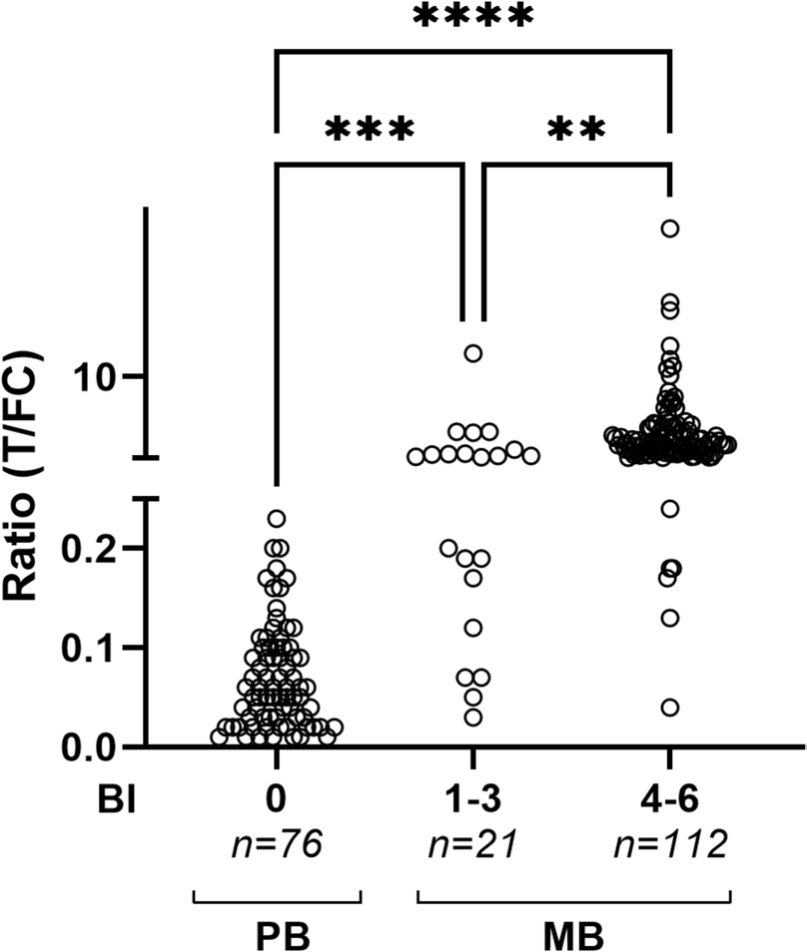
Anti-PGL-I IgM in PB and MB leprosy patients. Sera from PB (*n* = 76) and MB (*n* = 133) leprosy patients were examined by PGL-I QURapid. Leprosy patients were stratified for bacterial index (BI 0: *n* = 76; BI 1–3: *n* = 21; BI 4–6: *n* = 112). Ratio (R-)values (Y-axis) were calculated by dividing the peak area of the test line (T) by the peak area of the flow control line (FC). A Kruskal–Wallis test with Dunn’s correction for multiple testing was performed to determine the statistical significance between three groups (*P*-values: **P ≤ 0.01, ***P ≤ 0.001, ****P ≤ 0.0001)

PGL-I QURapid sensitivity was then assessed for 209 sera collected from leprosy patients with varying bacterial loads in Bangladesh. In total, 134 out of 209 individuals (64.1%) tested seropositive for anti-PGL-I IgM (cut-off R ≥ 0.16); 8 out of 76 PB patients (10.5%) and 126 out of 133 MB patients (94.7%) (Fig. 5). Patients with a BI of 1–3 and BI 4–6 had significantly increased anti-PGL-I IgM R-values compared to those with BI 0, with only seven testing seronegative (Kruskal–Wallis test; *P* ≤ 0.001 and *P* ≤ 0.0001, respectively). Anti-PGL-I IgM R-values were also significantly higher in MB patients with BI 4–6 compared to those with a BI of 1–3 (Kruskal–Wallis test; *P* ≤ 0.01).

### Anti-PGL-I IgM standard

**Fig. 6:**
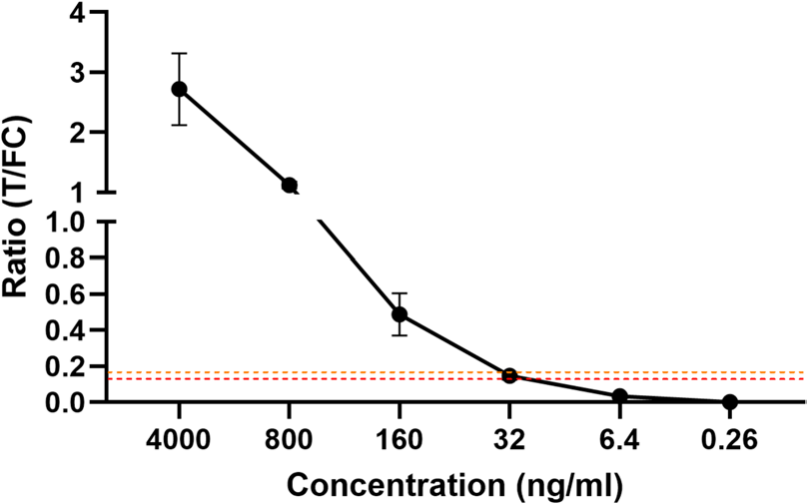
Recombinant anti-PGL-I IgM dilution series in buffer using PGL-I QURapid. A dilution series of the custom-made anti-PGL-I IgM was applied to the PGL-I QURapid. Each dilution was tested in triplicate. Ratio (R-)values (Y-axis) were calculated by dividing the peak area of the test line (T) by the peak area of the flow control line (FC). Mean values and standard deviations are shown. The orange and red dotted lines represent the cut-off for seropositivity (R ≥ 0.16) in serum and FSB (R ≥ 0.12), respectively

In view of quality assessment for the PGL-I QURapid (e.g. batch-to-batch comparison or monitoring different study sites using the same batch), humanized, recombinant anti-*M. leprae* PGL-I-specific IgM antibodies were custom-made. A standard dilution series of this recombinant anti-PGL-I IgM in buffer was applied to QUR-tests showing good performance (Fig. 6). The above determined cut-offs for seropositivity in serum (R ≥ 0.16) and FSB (R ≥ 0.12) corresponded to an antibody concentration of approximately 32 ng/ml.

## Discussion

Here, we present the PGL-I QURapid, which is an operational, quantitative rapid test for measurement of anti-PGL-I IgM levels in blood, permitting detection of past or present infection with *M. leprae*. This QUR-test shows excellent performance: its outcome is highly associated with bacillary load providing a 95% Sn for MB leprosy, while samples from patients with leishmaniasis or other mycobacterial diseases were all seronegative, indicating excellent specificity. Moreover, vaccination of healthy Dutch health care workers with live *M. bovis* BCG, did not affect the lack of anti-PGL-I seropositivity in this group.

### MB versus PB

These findings are in line with previous studies [11, 13, 14], and support the use of the PGL-I QURapid next to clinical assessment as an (adjunct) diagnostic test for MB leprosy and monitoring the effect of treatment [49, 50]. Inherent to PB leprosy is the very low number of bacteria in these patients that are only detectable by invasive biopsies. Thus, since the quantitative result of the PGL-I QURapid is associated with the number of bacteria, the here described low-invasive QUR-test could function as a discriminatory tool, applicable to distinguish MB from PB in settings where biopsies and their analysis are not easily performed. In this study, the QUR-test showed 10.5% Sn for PB patients. Therefore, for diagnosis of this patient group, detection of additional host biomarkers should be included. Of note is that this can also be accommodated on the UCP-LF platform, as was demonstrated for patients in Bangladesh, Brazil, China and Ethiopia [16, 17, 33]. In this respect, the development of multi-biomarker tests (MBTs) [33] is promising.

### Field-friendly, low-invasive and rapid test

The PGL-I QURapid is very well-suited for field settings as it only requires 20 µl of blood obtained by a finger prick. Furthermore, test results are visible within minutes after addition of sample to the QUR-test, and accurate quantitative values are available from 10 min onwards for both serum and FSB. This test-to-result time is well within the required optimal time window prescribed in the WHO TPP [31]. After use, the QUR-test can be stored indefinitely as a hard copy and rescanned when required. Inter- and intra-operator variability assessment demonstrated that results were highly reproducible thereby permitting utilization in field settings after limited training of local staff. In view of environmental considerations, the QUR-test was also tested successfully using plant-based biodegradable cassettes as a green solution for LFAs (Okos Diagnostics, the Netherlands, https://www.okosdiagnostics.com; data not shown).

### Test applications

A major benefit of a quantitative compared to a qualitative rapid test, is that one QUR-test can serve multiple use cases. Besides detection of infection on an individual level, another use case of the PGL-I QURapid is monitoring of recent *M. leprae* transmission in a population by serosurveys in healthy young children [10, 51]. This concept was piloted successfully with this format of the PGL-I QURapid in a serosurvey in a leprosy endemic area in India and was well accepted by health care staff as well as the targeted population [19]. In this respect, it is of note that assessment of sera of young children (*n* = 70) from a non-endemic area (the Netherlands) all resulted in R-values of zero ([19] and unpublished data), underlining the specificity of this QUR-test. Thus, based on the rationale that infection in young children reflects recent transmission in a population, this QUR-test can be applied to compare pediatric seroprevalence between different areas, as well as seroprevalence in a community before and after introduction of postexposure prophylactic interventions.

A skin disease that is endemic in areas where leprosy occurs, is leishmaniasis caused by *L. donovani* [52]. All leishmaniasis patients tested here were anti-PGL-I seronegative, emphasizing the high specificity of the PGL-I QURapid for *M. leprae* infection. Since leishmaniasis is included in the differential diagnosis of leprosy [30], developing a combined rapid test for the detection of infection with *M. leprae* and *L. donovani* could be both cost- and time-saving [52].

### Detecting infection in animals

Besides in humans, this QUR-test can also be used to detect *M. leprae* infection in different species as well as infection with *M. lepromatosis.* Previous research from our group has shown that the PGL-I QURapid can be applied to nine-banded armadillos and Eurasian red squirrels [18, 27, 53]. Also, we have recently applied the QUR-test to earprick samples of ten experimentally infected live armadillos showing high correlation with infection (in collaboration with the National Hansen’s Disease Program, USA; data not shown). Moreover, in agreement with what has been reported for ELISAs by Avanzi et al. [54], our QUR-test was able to detect anti-PGL-I antibodies in samples from *M. lepromatosis* infected squirrels [18] as well as a human case with this infection (data not shown).

### Standardization and quality control

The humanized, recombinant anti-PGL-I IgM control introduced in this study as part of the QUR-test kit, provides an essential tool to indicate thresholds for specific applications (sample type and use case). It offers high flexibility for manufacturing as test thresholds can be evaluated with a control sample of known IgM concentration. Production lots can be provided with predetermined cut-off R-values and UCP-LF strip readers eventually could be provided with standard curves calculating actual concentrations rather than R-values. This will allow improved comparison of data obtained with the PGL-I QURapid at different field sites or at different points in time.

### Limitation of the study

A limitation of this study was the low numbers available of serum samples from patients with TB, BU, leishmaniasis and NTMs from areas that are non-endemic for leprosy and from patients infected with *M. lepromatosis*, as well as FSB samples from leprosy patients. Future studies testing higher sample numbers with this QUR-test should be performed to validate our findings.

## Conclusions

The PGL-I QURapid for detection of *M. leprae* infection is operational and applicable for widescale use in leprosy research. The reported findings support production and distribution of this QUR-test with predetermined thresholds for seropositivity, set according to use case and sample type. Test results are operator-independent and performance over time can be monitored using humanized, recombinant anti-PGL-I IgM. The test protocol is straightforward, requires zero infrastructure, allowing application in POC settings although a UCP-compatible reader is required. As the test provides a quantitative result that can also be translated into a qualitative result, the PGL-I QURapid can be employed for various use cases in leprosy research and control programs.

## Supplementary Information

Supplementary Material 1. Figure 1: Effect of flow time on T and FC signals obtained with the PGL-I QURapid. Table 1: Overview of tests developed for the measurement of anti-PGL-I antibodies. Table 2: Cut-off determination according to Youden’s index and WHO TPP; based on MB leprosy vs NEC serum samples. Table 3: Cut-off determination according to Youden’s index and WHO TPP; based on MB leprosy vs NEC FSB samples. Table 4: PGL-I QURapid inter- and intra-operator variability

## Data Availability

The datasets used and/or analyzed during the current study are available from the corresponding author on reasonable request.

## Abbreviations

AUC: Area under the curve
BI: Bacterial index
BU: Buruli ulcer
FC line: Flow control line
FSB: Fingerstick blood
high: Anti-PGL-I IgM highly seropositive serum sample
IgM: Immunoglobulin M
LFA: Lateral flow assay
MB: Multibacillary
MDT: Multidrug therapy
med: Anti-PGL-I IgM medium serum sample
*M. leprae*: *Mycobacterium leprae*
*M. lepromatosis*: *Mycobacterium lepromatosis*
NEC: Non-endemic control
neg: Anti-PGL-I IgM seronegative serum sample
NTD: Neglected tropical disease
NTM: Non-tuberculous mycobacteria
Op A: Operator A
Op B: Operator B
Op C: Operator C
O/N: Overnight
PB: Paucibacillary
PGL-I: Phenolic glycolipid I
POC: Point-of-care/point-of-contact
QUR-test: Quantitative UCP-based rapid test
R: Ratio
RFU: Relative fluorescence unit
ROC: Receiver operating characteristic
Sn: Sensitivity
Sp: Specificity
TB: Tuberculosis
TFCEL: Task Force on definitions, criteria and indicators for interruption of transmission and elimination of leprosy
T line: Test line
TPP: Target product profile
UCP: Up-converting reporter particles
WHO: World Health Organization

## References

[1] Scollard DM, Adams LB, Gillis TP, Krahenbuhl JL, Truman RW, Williams DL. The continuing challenges of leprosy. Clin Microbiol Rev. 2006;19(2):338–81. 16614253 10.1128/CMR.19.2.338-381.2006 PMC1471987

[2] Deps P, Collin SM. *Mycobacterium**lepromatosis* as a second agent of Hansen’s disease. Front Microbiol. 2021;12: 698588. 34566911 10.3389/fmicb.2021.698588 PMC8461103

[3] Geluk A. Correlates of immune exacerbations in leprosy. Semin Immunol. 2018;39:111–8. 29950273 10.1016/j.smim.2018.06.003

[4] Blok DJ, De Vlas SJ, Richardus JH. Global elimination of leprosy by 2020: are we on track? Parasit Vectors. 2015;8:548. 26490878 10.1186/s13071-015-1143-4 PMC4618543

[5] Chen KH, Lin CY, Su SB, Chen KT. Leprosy: a review of epidemiology, clinical diagnosis, and management. J Trop Med. 2022;2022:8652062. 35832335 10.1155/2022/8652062 PMC9273393

[6] Towards Zero Leprosy. Global Leprosy (Hansen’s disease) Strategy 2021–2030. New Delhi: World Health Organization, Regional Office for South-East Asia; 2017.

[7] Smith WCS, Aerts A. Role of contact tracing and prevention strategies in the interruption of leprosy transmission. Leprosy Rev. 2014;85(1):2–17. 24974438

[8] Sales AM, de Leon AP, Düppre NC, Hacker MA, Nery JAC, Sarno EN, et al. Leprosy among patient contacts: a multilevel study of risk factors. Plos Neglect Trop D. 2011;5(3): e1013. 10.1371/journal.pntd.0001013 PMC3057944 21423643

[9] Belachew WA, Naafs B. Position statement: LEPROSY: diagnosis, treatment and follow-up. J Eur Acad Dermatol. 2019;33(7):1205–13. 10.1111/jdv.15569 30945360

[10] Pierneef L, van Hooij A, Taal A, Rumbaut R, Nobre ML, van Brakel W, et al. Detection of anti-*M.**leprae* antibodies in children in leprosy-endemic areas: a systematic review. PLoS Negl Trop Dis. 2021;15(8): e0009667. 34449763 10.1371/journal.pntd.0009667 PMC8428563

[11] van Hooij A, Tjon Kon Fat EM, van den Eeden SJF, Wilson L, Batista da Silva M, Salgado CG, et al. Field-friendly serological tests for determination of *M.**leprae*-specific antibodies. Sci Rep. 2017;7(1):8868. 28827673 10.1038/s41598-017-07803-7 PMC5566372

[12] TiemiNagao-Dias A, Casimiro de Macedo A, Rodrigues RO, Pedroza FHC, Albuquerque AA, Moreira FA, et al. Serum anti-PGL-1 IgG, IgM, and IgA in a 3-year follow-up study of 4–15-year-old leprosy contacts. Pediatr Infect Dis J. 2019;38(9):e193–8. 31220042 10.1097/INF.0000000000002337

[13] Tio-Coma M, Avanzi C, Verhard EM, Pierneef L, van Hooij A, Benjak A, et al. Genomic characterization of *Mycobacterium**lepraeto* explore transmission patterns identifies new subtype in Bangladesh. Front Microbiol. 2020;11:1220. 32612587 10.3389/fmicb.2020.01220 PMC7308449

[14] Zenha EM, Ferreira MA, Foss NT. Use of anti-PGL-1 antibodies to monitor therapy regimes in leprosy patients. Braz J Med Biol Res. 2009;42(10):968–72. 19784481 10.1590/s0100-879x2009001000016

[15] van Hooij A, Tjon Kon Fat EM, Richardus R, van den Eeden SJ, Wilson L, de Dood CJ, et al. Quantitative lateral flow strip assays as user-friendly tools to detect biomarker profiles for leprosy. Sci Rep. 2016;6:34260. 27682181 10.1038/srep34260 PMC5041085

[16] van Hooij A, Fat EMTK, da Silva MB, Bouth RC, Messias ACC, Gobbo AR, et al. Evaluation of immunodiagnostic tests for leprosy in Brazil, China and Ethiopia. Sci Rep. 2018;8: 17920. 30560920 10.1038/s41598-018-36323-1 PMC6298962

[17] van Hooij A, van den Eeden S, Richardus R, Fat ETK, Wilson L, Franken KLMC, et al. Application of new host biomarker profiles in quantitative point-of-care tests facilitates leprosy diagnosis in the field. EBioMedicine. 2019;47:301–8. 31422044 10.1016/j.ebiom.2019.08.009 PMC6796558

[18] Zhou Z, van Hooij A, Wassenaar GN, Seed E, Verhard-Seymonsbergen EM, Corstjens P, et al. Molecular and serological surveillance for *Mycobacterium**leprae* and *Mycobacterium**lepromatosis* in Wild Red Squirrels (*Sciurus**vulgaris*) from Scotland and Northern England. Animals (Basel). 2024;14(13):2005. 38998117 10.3390/ani14132005 PMC11240566

[19] Pierneef L, Malaviya P, van Hooij A, Sundar S, Singh AK, Kumar R, et al. Field-friendly anti-PGL-I serosurvey in children to monitor transmission in Bihar, India. Front Med. 2023;10:1260375. 10.3389/fmed.2023.1260375 PMC10565223 37828950

[20] Interruption of transmission and elimination of leprosy disease—technical guidance. New Delhi: World Health Organization, Regional Office for South-East Asia; 2023.

[21] van Dijk JHM, van Hooij A, Groot LM, Geboers J, Moretti R, Verhard-Seymonsbergen E, et al. Synthetic phenolic glycolipids for application in diagnostic tests for leprosy. ChemBioChem. 2021;22(8):1487–93. 33332701 10.1002/cbic.202000810 PMC8248333

[22] Duthie MS, Balagon MF, Maghanoy A, Orcullo FM, Cang M, Dias RF, et al. Rapid quantitative serological test for detection of infection with *Mycobacterium**leprae*, the causative agent of leprosy. J Clin Microbiol. 2014;52(2):613–9. 24478496 10.1128/JCM.02085-13 PMC3911347

[23] Duthie MS, Orcullo FM, Abbelana J, Maghanoy A, Balagon MF. Comparative evaluation of antibody detection tests to facilitate the diagnosis of multibacillary leprosy. Appl Microbiol Biotechnol. 2016;100(7):3267–75. 26820649 10.1007/s00253-016-7328-8

[24] Buhrer SS, Smits HL, Gussenhoven GC, van Ingen CW, Klatser PR. A simple dipstick assay for the detection of antibodies to phenolic glycolipid-I of *Mycobacterium**leprae*. Am J Trop Med Hyg. 1998;58(2):133–6. 9502593 10.4269/ajtmh.1998.58.133

[25] Buhrer-Sekula S, Smits HL, Gussenhoven GC, van Leeuwen J, Amador S, Fujiwara T, et al. Simple and fast lateral flow test for classification of leprosy patients and identification of contacts with high risk of developing leprosy. J Clin Microbiol. 2003;41(5):1991–5. 12734239 10.1128/JCM.41.5.1991-1995.2003 PMC154748

[26] Corstjens P, van Hooij A, Tjon Kon Fat EM, Alam K, Vrolijk LB, Dlamini S, et al. Fingerstick test quantifying humoral and cellular biomarkers indicative for *M.**leprae* infection. Clin Biochem. 2019;66:76–82. 30695682 10.1016/j.clinbiochem.2019.01.007

[27] Zhou Z, Pena M, van Hooij A, Pierneef L, de Jong D, Stevenson R, et al. Detection and monitoring of *Mycobacterium**leprae* infection in nine banded armadillos (*Dasypus**novemcinctus*) using a quantitative rapid test. Front Microbiol. 2021;12: 763289. 34777319 10.3389/fmicb.2021.763289 PMC8581735

[28] Niedbala RS, Feindt H, Kardos K, Vail T, Burton J, Bielska B, et al. Detection of analytes by immunoassay using up-converting phosphor technology. Anal Biochem. 2001;293(1):22–30. 11373074 10.1006/abio.2001.5105

[29] Corstjens PL, Li S, Zuiderwijk M, Kardos K, Abrams WR, Niedbala RS, et al. Infrared up-converting phosphors for bioassays. IEE Proc Nanobiotechnol. 2005;152(2):64–72. 16441160 10.1049/ip-nbt:20045014

[30] Trindade MA, Silva LL, Braz LM, Amato VS, Naafs B, Sotto MN. Post-kala-azar dermal leishmaniasis and leprosy: case report and literature review. Bmc Infect Dis. 2015;15:543. 26592919 10.1186/s12879-015-1260-x PMC4656188

[31] World Health Organization. Target product profile for a diagnostic test to detect *Mycobacterium leprae* infection among asymptomatic household and familial contacts of leprosy patients. World Health Organization; 2023.

[32] Richardus RA, Alam K, Pahan D, Feenstra SG, Geluk A, Richardus JH. The combined effect of chemoprophylaxis with single dose rifampicin and immunoprophylaxis with BCG to prevent leprosy in contacts of newly diagnosed leprosy cases: a cluster randomized controlled trial (MALTALEP study). Bmc Infect Dis. 2013;13:1–8. 24088534 10.1186/1471-2334-13-456 PMC3850918

[33] van Hooij A, Fat EMTK, de Jong D, Khatun M, Soren S, Chowdhury A, et al. Prototype multi-biomarker test for point-of-care leprosy diagnostics. Iscience. 2021;24(1): 102006. 33490914 10.1016/j.isci.2020.102006 PMC7807156

[34] Ridley DS, Jopling WH. Classification of leprosy according to immunity. A five-group system. Int J Lepr Other Mycobact Dis. 1966;34(3):255–73. 5950347

[35] Lin MY, Geluk A, Smith SG, Stewart AL, Friggen AH, Franken KL, et al. Lack of immune responses to *Mycobacterium**tuberculosis* DosR regulon proteins following *Mycobacterium**bovis* BCG vaccination. Infect Immun. 2007;75(7):3523–30. 17502400 10.1128/IAI.01999-06 PMC1932964

[36] Leyten EMS, Lin MY, Franken KLMC, Friggen AH, Prins C, van Meijgaarden KE, et al. Human T-cell responses to 25 novel antigens encoded by genes of the dormancy regulon of *Mycobacterium**tuberculosis*. Microbes Infect. 2006;8(8):2052–60. 16931093 10.1016/j.micinf.2006.03.018

[37] Wortmann G, Sweeney C, Houng HS, Aronson N, Stiteler J, Jackson J, et al. Rapid diagnosis of leishmaniasis by fluorogenic polymerase chain reaction. Am J Trop Med Hyg. 2001;65(5):583–7. 11716118 10.4269/ajtmh.2001.65.583

[38] Kuilder JS, Wismans PJ, Baerveldt EM, van Hellemond JJ, Melo MD, van Genderen PJJ. Cutaneous leishmaniasis in 3 travelers returning from Israel to the Netherlands. Emerg Infect Dis. 2016;22(11):2022–4. 27767930 10.3201/eid2211.161154 PMC5088023

[39] Bibert S, Bratschi MW, Aboagye SY, Collinet E, Scherr N, Yeboah-Manu D, et al. Susceptibility to *Mycobacterium**ulcerans* disease (Buruli ulcer) is associated with *IFNG* and *iNOS* gene polymorphisms. Front Microbiol. 2017;8:1903. 29046669 10.3389/fmicb.2017.01903 PMC5632961

[40] World Health Organization. Treatment of *Mycobacterium**ulcerans* disease (Buruli Ulcer): guidance for health workers. Geneva: World Health Organization; 2012.

[41] Haverkamp MH, van Wengen A, de Visser AW, van Kralingen KW, van Dissel JT, van de Vosse E. Pulmonary: a canary in the cystic fibrosis coalmine. J Infect. 2012;64(6):609–12. 22366207 10.1016/j.jinf.2012.02.010

[42] Kilic SS, van Wengen A, de Paus RA, Celebi S, Meziane B, Hafizoglu D, et al. Severe disseminated mycobacterial infection in a boy with a novel mutation leading to IFN-γR2 deficiency. J Infect. 2012;65(6):568–72. 22902943 10.1016/j.jinf.2012.08.008

[43] Potjewijd J, de Paus RA, van Wengen A, Damoiseaux J, Verbon A, van de Vosse E. Disseminated infection in a patient with a novel partial interleukin-12/23 receptor β1 deficiency. Clin Immunol. 2012;144(2):83–6. 22695533 10.1016/j.clim.2012.05.007

[44] Arend SM, de Palou EC, de Haas P, Janssen R, Hoeve MA, Verhard EM, et al. Pneumonia caused by in a series of patients without recognised immune defect. Clin Microbiol Infect. 2004;10(8):738–48. 15301677 10.1111/j.1469-0691.2004.00898.x

[45] Ten Doesschate T, van der Vaart TW, Debisarun PA, Taks E, Moorlag S, Paternotte N, et al. Bacillus Calmette-Guerin vaccine to reduce healthcare worker absenteeism in COVID-19 pandemic, a randomized controlled trial. Clin Microbiol Infect. 2022;28(9):1278–85. 35489606 10.1016/j.cmi.2022.04.009 PMC9046133

[46] Pierneef L, van Hooij A, de Jong D, Tjon Kon Fat EM, van Meijgaarden KE, Petruccioli E, et al. Host biomarker-based quantitative rapid tests for detection and treatment monitoring of tuberculosis and COVID-19. iScience. 2023;26(1): 105873. 36590898 10.1016/j.isci.2022.105873 PMC9791715

[47] Fluss R, Faraggi D, Reiser B. Estimation of the Youden index and its associated cutoff point. Biometrical J. 2005;47(4):458–72. 10.1002/bimj.200410135 16161804

[48] Mokkapati VK, Sam Niedbala R, Kardos K, Perez RJ, Guo M, Tanke HJ, et al. Evaluation of UPlink-RSV: prototype rapid antigen test for detection of respiratory syncytial virus infection. Ann N Y Acad Sci. 2007;1098:476–85. 17435154 10.1196/annals.1384.021

[49] Corstjens P, van Hooij A, Tjon Kon EM, van den Eeden SJF, Wilson L, Geluk A. Field-friendly test for monitoring multiple immune response markers during onset and treatment of exacerbated immunity in leprosy. Clin Vaccine Immunol. 2016;23(6):515–9. 27030588 10.1128/CVI.00033-16 PMC4895013

[50] Khadge S, Banu S, Bobosha K, van der Ploeg-van Schip JJ, Goulart IM, Thapa P, et al. Longitudinal immune profiles in type 1 leprosy reactions in Bangladesh, Brazil, Ethiopia and Nepal. Bmc Infect Dis. 2015;15:477. 26510990 10.1186/s12879-015-1128-0 PMC4625471

[51] Zhou Z, Pierneef L, van Hooij A, Geluk A. Detection of anti-*M.**leprae* antibodies in healthy children in China: a systematic review of Chinese literature. Front Trop Dis. 2022;3: 963674.

[52] Cloots K, Uranw S, Ostyn B, Bhattarai NR, Le Rutte E, Khanal B, et al. Impact of the visceral leishmaniasis elimination initiative on *Leishmania**donovani* transmission in Nepal: a 10-year repeat survey. Lancet Glob Health. 2020;8(2):e237–43. 31981555 10.1016/S2214-109X(19)30536-4

[53] Schilling AK, McCurdy K, Fish A, Lurz PWW, Geluk A, Van Hooij A, et al. Diagnosing and categorizing leprosy in live Eurasian red squirrels (*Sciurus vulgaris*) for management, surveillance, and translocation purposes. J Zoo Wildl Med. 2021;52(2):648–59. 34130408 10.1638/2020-0066

[54] Avanzi C, Del-Pozo J, Benjak A, Stevenson K, Simpson VR, Busso P, et al. Red squirrels in the British Isles are infected with leprosy bacilli. Science. 2016;354(6313):744–7. 27846605 10.1126/science.aah3783

