# Supplementary material for: Rapid test for Mycobacterium leprae infection: a practical tool for leprosy
Source: Infect Dis Poverty. 2024 Dec 2;13:88. doi: 10.1186/s40249-024-01262-9 (PMC11610287; doi:10.1186/s40249-024-01262-9)
Supplement: Supplementary file 1 — Supplementary Material 1. Figure 1: Effect of flow time on T and FC signals obtained with the PGL-I QURapid. Table 1: Overview of tests developed for the measurement of anti-PGL-I antibodies. Table 2: Cut-off determination according to Youden’s index and WHO TPP; based on MB leprosy vs NEC serum samples. Table 3: Cut-off determination according to Youden’s index and WHO TPP; based on MB leprosy vs NEC FSB samples. Table 4: PGL-I QURapid inter- and intra-operator variability [file 40249_2024_1262_MOESM1_ESM.docx]

**Supplemental Information**


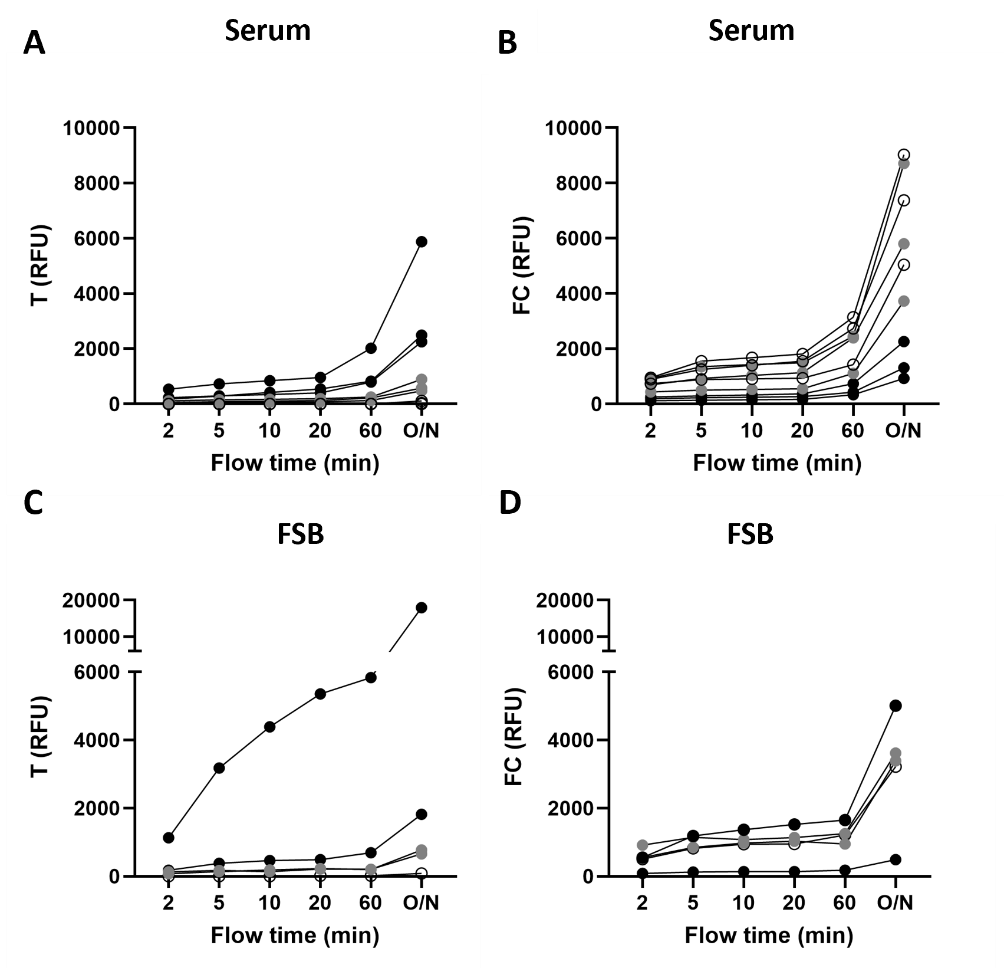


**Supplementary figure 1: Effect of flow time on T and FC signals obtained with the PGL-I QURapid.**

Anti-PGL-I IgM highly seropositive, medium and seronegative control sera (A-B) and FSB samples (C-D) were examined by the PGL-I QURapid. Samples were measured at 2, 5, 10, 20, 60 minutes and 24 hours (O/N) after sample addition. Black dots: anti-PGL-I IgM highly seropositive serum samples; grey dots: anti-PGL-I IgM medium serum samples; green dots: anti-PGL-I IgM seronegative serum samples.

A: T line values (Y-axis) in relative fluorescence units (RFU) as a function of serum sample flow time in minutes. B: FC line values (Y-axis) in RFU as a function of serum sample flow time in minutes.

C: T line values (Y-axis) in RFU as a function of FSB sample flow time in minutes. D: FC line values (Y-axis) in RFU as a function of FSB sample flow time in minutes.

| **Method** | **ref. no.** | **target** | **Ab** | **quantitative/**  **qualitative** | | **rapid (yes/no)** |
| --- | --- | --- | --- | --- | --- | --- |
| ELISA | 11, 21 | ND-O-HSA/NPT-H-BSA | IgM and IgG | | quantitative | no |
| OrangeLife NDO-LID RDT® | 22 | NDO-LID | IgM and IgG | | qualitative | yes |
| CTK OnSite Leprosy Ab Rapid Test | 11, 23 | PGL-I and LID-1 | IgM and IgG | | qualitative | yes |
| ML-dipstick | 24 | ND-O-BSA | IgM | | qualitative | yes |
| ML-flow | 25 | NT-P-BSA | IgM | | qualitative | yes |

**Supplementary Table 1: *Overview of tests developed for the measurement of anti-PGL-I antibodies.***

Test method, reference in which the test is described, test target, and antibody measured are listed. In addition, it is indicated whether tests are quantitative or qualitative and rapid or not. Ab: antibody; ELISA: enzyme-linked immunosorbent assay; IgG: immunoglobulin G; IgM: immunoglobulin M; LID-1: leprosy IDRI diagnostic-1; ND-O-BSA: natural disaccharide-octyl bovine serum albumin; ND-O-HSA: natural disaccharide-octyl human serum albumin; NDO-LID: natural disaccharide-octyl leprosy IDRI diagnostic; NPT-H-BSA: phenolic trisaccharide functionalized with a hexanoic acid linker for conjugation to bovine serum albumin; NT-P-BSA: natural trisaccharide phenyl bovine serum albumin; PGL-I: phenolic glycolipid I; RDT: rapid diagnostic test.

| **Supplementary Table 2: *Cut-off determination according to Youden’s index and WHO TPP; based on MB leprosy vs NEC serum samples.*** | | | | | |  |
| --- | --- | --- | --- | --- | --- | --- |
| **Cut-off** | **Sensitivity** | **95% *CI*** | **Specificity** | **95% *CI*** | **Youden’s index** | |
| > 0.02500 | 1 | 0.9719 to 1.000 | 0.7 | 0.6349 to 0.7579 | 0.70 | |
| > 0.03500 | 0.9925 | 0.9586 to 0.9996 | 0.7952 | 0.7356 to 0.8443 | 0.79 | |
| > 0.04500 | 0.985 | 0.9468 to 0.9973 | 0.8524 | 0.7981 to 0.8940 | 0.84 | |
| > 0.05500 | 0.9774 | 0.9358 to 0.9939 | 0.9 | 0.8520 to 0.9337 | 0.88 | |
| > 0.06500 | 0.9774 | 0.9358 to 0.9939 | 0.9238 | 0.8798 to 0.9526 | 0.90 | |
| > 0.07500 | 0.9624 | 0.9150 to 0.9838 | 0.9429 | 0.9028 to 0.9670 | 0.91 | |
| > 0.08500 | 0.9624 | 0.9150 to 0.9838 | 0.9476 | 0.9087 to 0.9705 | 0.91 | |
| > 0.09500 | 0.9624 | 0.9150 to 0.9838 | 0.9524 | 0.9146 to 0.9739 | 0.91 | |
| > 0.1050 | 0.9624 | 0.9150 to 0.9838 | 0.9619 | 0.9266 to 0.9806 | 0.92 | |
| > 0.1150 | 0.9624 | 0.9150 to 0.9838 | 0.9667 | 0.9328 to 0.9838 | 0.93 | |
| > 0.1250 | 0.9549 | 0.9051 to 0.9792 | 0.9762 | 0.9455 to 0.9898 | 0.93 | |
| > 0.1350 | 0.9474 | 0.8953 to 0.9743 | 0.9857 | 0.9588 to 0.9961 | 0.93 | |
| > 0.1450 | 0.9474 | 0.8953 to 0.9743 | 0.9905 | 0.9659 to 0.9983 | 0.94 | |
| *> 0.1600* | *0.9474* | *0.8953 to 0.9743* | *1* | *0.9820 to 1.000* | *0.95* | |
| > 0.1750 | 0.9323 | 0.8764 to 0.9640 | 1 | 0.9820 to 1.000 | 0.93 | |
| > 0.1850 | 0.9173 | 0.8580 to 0.9532 | 1 | 0.9820 to 1.000 | 0.92 | |
| > 0.1950 | 0.9023 | 0.8399 to 0.9420 | 1 | 0.9820 to 1.000 | 0.90 | |
| > 0.2200 | 0.8947 | 0.8311 to 0.9363 | 1 | 0.9820 to 1.000 | 0.89 | |
| > 0.2600 | 0.8872 | 0.8222 to 0.9305 | 1 | 0.9820 to 1.000 | 0.89 | |
| > 0.2900 | 0.8797 | 0.8135 to 0.9246 | 1 | 0.9820 to 1.000 | 0.88 | |
| > 0.3150 | 0.8722 | 0.8048 to 0.9186 | 1 | 0.9820 to 1.000 | 0.87 | |
| > 0.3400 | 0.8647 | 0.7962 to 0.9126 | 1 | 0.9820 to 1.000 | 0.86 | |
| > 0.3600 | 0.8571 | 0.7876 to 0.9066 | 1 | 0.9820 to 1.000 | 0.86 | |
| > 0.3850 | 0.8496 | 0.7791 to 0.9005 | 1 | 0.9820 to 1.000 | 0.85 | |
| > 0.4050 | 0.8421 | 0.7707 to 0.8943 | 1 | 0.9820 to 1.000 | 0.84 | |
| > 0.4350 | 0.8346 | 0.7622 to 0.8882 | 1 | 0.9820 to 1.000 | 0.83 | |
| > 0.4650 | 0.8271 | 0.7539 to 0.8819 | 1 | 0.9820 to 1.000 | 0.83 | |
| > 0.4900 | 0.8195 | 0.7455 to 0.8756 | 1 | 0.9820 to 1.000 | 0.82 | |
| > 0.5200 | 0.812 | 0.7372 to 0.8693 | 1 | 0.9820 to 1.000 | 0.81 | |
| > 0.5550 | 0.8045 | 0.7290 to 0.8630 | 1 | 0.9820 to 1.000 | 0.80 | |
| > 0.5900 | 0.797 | 0.7207 to 0.8566 | 1 | 0.9820 to 1.000 | 0.80 | |
| > 0.6150 | 0.7895 | 0.7126 to 0.8501 | 1 | 0.9820 to 1.000 | 0.79 | |
| > 0.6350 | 0.7744 | 0.6963 to 0.8372 | 1 | 0.9820 to 1.000 | 0.77 | |
| > 0.6700 | 0.7669 | 0.6882 to 0.8307 | 1 | 0.9820 to 1.000 | 0.77 | |
| > 0.7050 | 0.7594 | 0.6801 to 0.8241 | 1 | 0.9820 to 1.000 | 0.76 | |
| > 0.7200 | 0.7519 | 0.6721 to 0.8175 | 1 | 0.9820 to 1.000 | 0.75 | |
| > 0.7350 | 0.7444 | 0.6641 to 0.8109 | 1 | 0.9820 to 1.000 | 0.74 | |
| > 0.7750 | 0.7293 | 0.6482 to 0.7976 | 1 | 0.9820 to 1.000 | 0.73 | |
| > 0.8200 | 0.7218 | 0.6402 to 0.7909 | 1 | 0.9820 to 1.000 | 0.72 | |
| > 0.8350 | 0.7143 | 0.6323 to 0.7842 | 1 | 0.9820 to 1.000 | 0.71 | |
| > 0.8500 | 0.7068 | 0.6245 to 0.7775 | 1 | 0.9820 to 1.000 | 0.71 | |

Overview of the results from calculating a cut-off for anti-PGL-I IgM seropositivity in sera from healthy controls vs. MB leprosy patients, by using the Youden’s index and WHO TPP, including corresponding sensitivity and specificity (in italic red font). Note: Sn/Sp values are depicted as fractions. Only Sn/Sp values between 0.7 and 1.0 are shown.

| **Supplementary Table 3: *Cut-off determination according to Youden’s index and WHO TPP; based on MB leprosy vs NEC FSB samples.*** | | | | | | |
| --- | --- | --- | --- | --- | --- | --- |
| **Cut-off** | **Sensitivity** | **95% *CI*** | **Specificity** | **95% *CI*** | **Youden’s index** |  |
| > 0.02500 | 0.9787 | 0.8889 to 0.9989 | 0.784 | 0.7458 to 0.8178 | 0.7627 |  |
| > 0.03500 | 0.9574 | 0.8575 to 0.9924 | 0.872 | 0.8399 to 0.8985 | 0.8294 |  |
| > 0.04500 | 0.9574 | 0.8575 to 0.9924 | 0.932 | 0.9065 to 0.9509 | 0.8894 |  |
| *> 0.05500* | *0.9574* | *0.8575 to 0.9924* | *0.962* | *0.9414 to 0.9755* | *0.9194* |  |
| > 0.06500 | 0.9362 | 0.8284 to 0.9781 | 0.966 | 0.9462 to 0.9787 | 0.9022 |  |
| > 0.07500 | 0.9149 | 0.8007 to 0.9664 | 0.976 | 0.9585 to 0.9862 | 0.8909 |  |
| > 0.08500 | 0.9149 | 0.8007 to 0.9664 | 0.986 | 0.9714 to 0.9932 | 0.9009 |  |
| > 0.09500 | 0.9149 | 0.8007 to 0.9664 | 0.992 | 0.9796 to 0.9969 | 0.9069 |  |
| > 0.1050 | 0.9149 | 0.8007 to 0.9664 | 0.994 | 0.9825 to 0.9984 | 0.9089 |  |
| **> 0.1200** | **0.9149** | **0.8007 to 0.9664** | **0.998** | **0.9888 to 0.9999** | **0.9129** |  |
| > 0.1350 | 0.8723 | 0.7483 to 0.9402 | 0.998 | 0.9888 to 0.9999 | 0.8703 |  |
| > 0.1450 | 0.8511 | 0.7231 to 0.9259 | 0.998 | 0.9888 to 0.9999 | 0.8491 |  |
| > 0.1650 | 0.8298 | 0.6986 to 0.9111 | 0.998 | 0.9888 to 0.9999 | 0.8278 |  |
| > 0.2000 | 0.8085 | 0.6746 to 0.8958 | 0.998 | 0.9888 to 0.9999 | 0.8065 |  |
| > 0.2350 | 0.7872 | 0.6510 to 0.8801 | 0.998 | 0.9888 to 0.9999 | 0.7852 |  |
| > 0.2750 | 0.766 | 0.6278 to 0.8640 | 0.998 | 0.9888 to 0.9999 | 0.764 |  |
| > 0.3300 | 0.7234 | 0.5824 to 0.8306 | 0.998 | 0.9888 to 0.9999 | 0.7214 |  |
| > 0.3900 | 0.7234 | 0.5824 to 0.8306 | 1 | 0.9924 to 1.000 | 0.7234 |  |
| > 0.4300 | 0.7021 | 0.5602 to 0.8135 | 1 | 0.9924 to 1.000 | 0.7021 |  |

Overview of the results from calculating a cut-off for anti-PGL-I IgM seropositivity in FSB samples from healthy controls vs. MB leprosy patients, by using the Youden’s index (in italic red font) and the WHO TPP (in bold), including corresponding sensitivity and specificity. Note: Sn/Sp values are depicted as fractions. Only Sn/Sp values between 0.7 and 1.0 are shown.

**Supplementary Table 4:** ***PGL-I QURapid inter- and intra-operator variability.***

| **Control sample** | **High 1** | | | **High 2** | | | **Med** | | | **Neg 1** | | | **Neg 2** | | |
| --- | --- | --- | --- | --- | --- | --- | --- | --- | --- | --- | --- | --- | --- | --- | --- |
|  | **Ratio** | | | | | | | | | | | | | | |
| Operator | A | B | C | A | B | C | A | B | C | A | B | C | A | B | C |
| Day 1 | 1.76 | 1.97 | 1.82 | 2.34 | 3.76 | 2.53 | 0.14 | 0.13 | 0.12 | 0.01 | 0.01 | 0.01 | 0.00 | 0.00 | 0.00 |
|  | 2.30 | 1.68 | 2.39 | 3.35 | 2.98 | 2.02 | 0.11 | 0.14 | 0.10 | 0.01 | 0.01 | 0.00 | 0.01 | 0.01 | 0.00 |
|  | 2.55 | 1.83 | 2.02 | 2.56 | 2.69 | 2.64 | 0.12 | 0.10 | 0.09 | 0.01 | 0.00 | 0.01 | 0.00 | 0.01 | 0.00 |
| Mean of triplicate | 2.20 | 1.83 | 2.08 | 2.75 | 3.14 | 2.40 | 0.12 | 0.12 | 0.10 | 0.01 | 0.01 | 0.01 | 0.00 | 0.01 | 0.00 |
| SD of triplicate | 0.40 | 0.15 | 0.29 | 0.53 | 0.55 | 0.33 | 0.02 | 0.02 | 0.02 | 0.00 | 0.01 | 0.01 | 0.01 | 0.01 | 0.00 |
| SD between operators | 0.19 | | | 0.37 | | | 0.01 | | | 0.00 | | | 0.00 | | |
| Day 2 | 1.81 | 1.94 | 2.34 | 3.89 | 2.75 | 2.38 | 0.14 | 0.14 | 0.19 | 0.00 | 0.00 | 0.01 | 0.00 | 0.01 | 0.00 |
|  | 2.08 | 2.16 | 1.82 | 2.85 | 2.51 | 3.19 | 0.12 | 0.13 | 0.15 | 0.01 | 0.00 | 0.01 | 0.00 | 0.01 | 0.00 |
|  | 2.50 | 1.86 | 1.96 | 2.09 | 3.33 | 2.37 | 0.12 | 0.14 | 0.13 | 0.01 | 0.01 | 0.01 | 0.00 | 0.01 | 0.01 |
| Mean of triplicate | 2.13 | 1.99 | 2.04 | 2.94 | 2.86 | 2.65 | 0.13 | 0.14 | 0.16 | 0.01 | 0.00 | 0.01 | 0.00 | 0.01 | 0.00 |
| SD of triplicate | 0.35 | 0.16 | 0.27 | 0.90 | 0.42 | 0.47 | 0.01 | 0.01 | 0.03 | 0.01 | 0.01 | 0.00 | 0.00 | 0.00 | 0.01 |
| SD between operators | 0.07 | | | 0.15 | | | 0.02 | | | 0.00 | | | 0.01 | | |
| Day 3 | 2.09 | 2.00 | 2.04 | 2.22 | 3.48 | 2.60 | 0.17 | 0.11 | 0.10 | 0.00 | 0.01 | 0.01 | 0.02 | 0.01 | 0.00 |
|  | 2.26 | 2.32 | 2.10 | 2.53 | 4.24 | 2.42 | 0.12 | 0.14 | 0.13 | 0.02 | 0.00 | 0.01 | 0.00 | 0.00 | 0.00 |
|  | 2.15 | 2.26 | 1.53 | 2.70 | 2.50 | 1.90 | 0.15 | 0.17 | 0.10 | 0.00 | 0.01 | 0.00 | 0.00 | 0.00 | 0.01 |
| Mean of triplicate | 2.17 | 2.19 | 1.89 | 2.48 | 3.41 | 2.31 | 0.15 | 0.14 | 0.11 | 0.01 | 0.01 | 0.01 | 0.01 | 0.00 | 0.00 |
| SD of triplicate | 0.09 | 0.17 | 0.31 | 0.24 | 0.87 | 0.36 | 0.03 | 0.03 | 0.02 | 0.01 | 0.01 | 0.01 | 0.01 | 0.01 | 0.01 |
| SD between operators | 0.17 | | | 0.59 | | | 0.02 | | | 0.00 | | | 0.00 | | |
| Mean of all days | 2.17 | 2.00 | 2.00 | 2.73 | 3.14 | 2.45 | 0.13 | 0.13 | 0.12 | 0.01 | 0.01 | 0.01 | 0.00 | 0.01 | 0.00 |
| SD of all days | 0.04 | 0.18 | 0.10 | 0.23 | 0.27 | 0.18 | 0.01 | 0.01 | 0.03 | 0.00 | 0.00 | 0.00 | 0.00 | 0.00 | 0.00 |

Overview of the results from testing the PGL-I QURapid robustness and reproducibility using anti-PGL-I IgM highly seropositive (High 1-2), medium (Med) and seronegative (Neg 1-2) control serum samples by three operators on three different days. Results are shown per operator (A-C), day (1-3) and control sample. Mean and standard deviation (SD) are shown.
